# The Structure–Antiproliferative Activity Relationship of Pyridine Derivatives

**DOI:** 10.3390/ijms25147640

**Published:** 2024-07-11

**Authors:** Ana-Laura Villa-Reyna, Martin Perez-Velazquez, Mayra Lizett González-Félix, Juan-Carlos Gálvez-Ruiz, Dulce María Gonzalez-Mosquera, Dora Valencia, Manuel G. Ballesteros-Monreal, Milagros Aguilar-Martínez, Mario-Alberto Leyva-Peralta

**Affiliations:** 1 Departamento de Ciencias Químico Biológicas y Agropecuarias, Facultad Interdisiplinaria de Ciencias Biológicas y de Salud, Universidad de Sonora, Campus Caborca, Caborca 83600, Mexico; ana.villa@unison.mx (A.-L.V.-R.); dora.valencia@unison.mx (D.V.); manuel.ballesteros@unison.mx (M.G.B.-M.); 2 Departamento de Investigaciones Científicas y Tecnológicas, Facultad Interdisiplinaria de Ciencias Biológicas y de Salud, Universidad de Sonora, Campus Hermosillo, Hermosillo 83000, Mexico; martin.perez@unison.mx (M.P.-V.); mayra.gonzalez@unison.mx (M.L.G.-F.); 3 Departamento de Ciencias Químico Biológicas, Facultad Interdisiplinaria de Ciencias Biológicas y de Salud, Universidad de Sonora, Campus Hermosillo, Hermosillo 83000, Mexico; juan.galvez@unison.mx; 4 Departamento de Farmacia, Facultad de Química-Farmacia, Universidad Central Marta Abreu Las Villitas, Santa Clara, Cuba; dgonzalezmosquera@gmail.com

**Keywords:** pyridine, derivative, biological activity, antiproliferative, structure–activity

## Abstract

Pyridine, a compound with a heterocyclic structure, is a key player in medicinal chemistry and drug design. It is widely used as a framework for the design of biologically active molecules and is the second most common heterocycle in FDA-approved drugs. Pyridine is known for its diverse biological activity, including antituberculosis, antitumor, anticoagulant, antiviral, antimalarial, antileishmania, anti-inflammatory, anti-Alzheimer’s, antitrypanosomal, antimalarial, vasodilatory, antioxidant, antimicrobial, and antiproliferative effects. This review, spanning from 2022 to 2012, involved the meticulous identification of pyridine derivatives with antiproliferative activity, as indicated by their minimum inhibitory concentration values (IC50) against various cancerous cell lines. The aim was to determine the most favorable structural characteristics for their antiproliferative activity. Using computer programs, we constructed and calculated the molecular descriptors and analyzed the electrostatic potential maps of the selected pyridine derivatives. The study found that the presence and positions of the -OMe, -OH, -C=O, and NH2 groups in the pyridine derivatives enhanced their antiproliferative activity over the cancerous cellular lines studied. Conversely, pyridine derivatives with halogen atoms or bulky groups in their structures exhibited lower antiproliferative activity.

## 1. Introduction

Cancer is a chronic disease that is among the three leading causes of death worldwide; it accounted for around 20 million deaths in 2020 (one in every six deaths globally). It is estimated that the number of cases will increase to around 37 million by 2040 [1,2,3]. The most prevalent types of cancer include breast, lung, prostate, skin, stomach, colon, and rectal cancer [3].

The main treatments against cancer are chemotherapy, radiotherapy, and surgery. Although these treatments have evolved, they face several challenges [4]. Chemotherapy remains the main treatment due to its effectiveness in eliminating cancer cells, although it has also been associated with significant side effects [5,6].

Multidrug resistance (MDR) is a growing public health problem worldwide and is one of the main challenges facing cancer research, as it leads to the inefficiency of current therapies [7]. Currently, cancer cells are compared to superbacteria, since it has been shown that 50% of cancer cells are resistant to all anticancer drugs used [7]. A specific part of cancer research has focused on the search for new alternatives to combat the problem of drug resistance to improve the clinical outcomes, because there are also options that are selective against cancer cells and do not affect healthy cells [7,8].

The design and synthesis of molecules for cancer treatment that improve the characteristics of clinical drugs enable the establishment of new anticancer agents with promising activity [3]. Structure–activity relationship studies have been used as a strategy to develop new anticancer agents to inhibit the growth of cancer cells [1]. The main concern in the structure-based drug design approach has been the selection of molecular structures to develop pharmacologically active agents. Nitrogen-containing heterocycle compounds have been widely identified for their anticancer activity and are a scaffold of great interest for the design of these pharmacological alternatives [3].

Due to their structural and chemical diversity, heterocycles have been considered promising scaffolds in rational design for the construction of new biologically active molecules. The advantage of heterocyclic chemistry is the number of available routes to modify their structures in the laboratory—for example, changing the type and number of heteroatoms or the size of the ring or incorporating various functional groups as substituents [8]. Such structures are found in more than 90% of newly synthesized drugs, with outstanding pharmacological, pharmacokinetic, and toxicological properties [9].

Nitrogen-containing heterocycles have been the subject of numerous organic and medicinal chemistry studies. These compounds are found in both natural and synthetic molecules and exhibit a diverse range of biological activity [10]. Some of the most well known heterocycles of this type include benzimidazoles, benzothiazoles, benzoxazoles, imidazopyridines, pyridines, and aminopyridines [10].

Pyridine is the second most common heterocycle found in FDA-approved drugs in the United States of America. It is similar to benzene regarding its planarity characteristics and the ability to perform pi interactions. Pyridine is formed by replacing one carbon atom with nitrogen, which changes its molecular and physicochemical properties. This modification modulates its lipophilicity and improves its aqueous solubility, metabolic stability, and ability to form hydrogen bonds [11].

There have been reports of a wide range of pyridine-derived compounds that possess diverse biological activity. This activity includes antituberculosis [12,13,14], antitumor [15,16,17,18,19,20,21,22,23], anticoagulant [24], antiviral [25,26,27], antimalarial [28], antileishmanial [29], anti-inflammatory [30], anti-Alzheimer’s [31,32], antitrypanosomal [33,34,35], vasodilating [36], antioxidant [37,38], antimicrobial [38,39,40,41,42,43,44,45,46,47,48,49,50,51,52,53,54], and antiproliferative [55,56,57,58,59,60,61,62,63,64,65,66] properties. This review aims to analyze articles published from 2012 to 2022 and identify the structural modifications that can enhance the antiproliferative activity of pyridine derivatives on various cancerous cell lines.

## 2. Results

**Figure 1:**
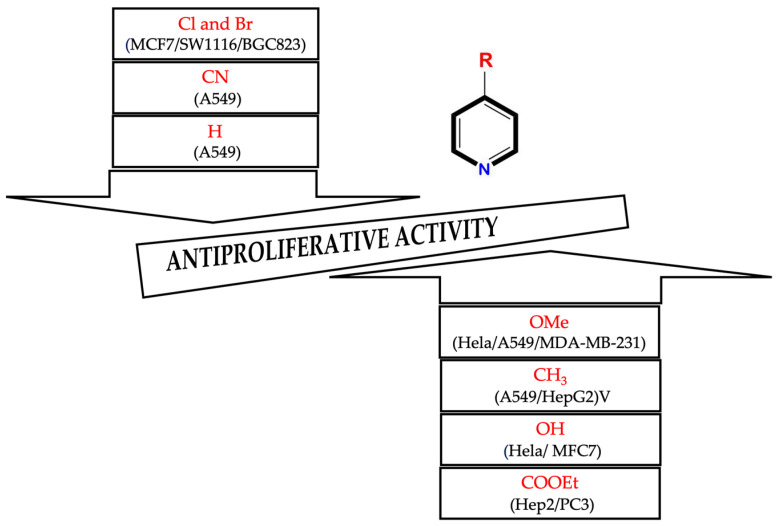
List of functional groups identified in pyridine-derived compounds that increase or decrease their antiproliferative activity in different cancer cell lines.

In this review, a search was carried out in the literature for antiproliferative activity studies of various compounds synthesized from pyridine as the central scaffold, and a structure–activity relationship analysis was carried out to identify which functional groups or structural modifications are related to better antiproliferative activity against various human cancer cell lines. Figure 1 summarizes the analysis that was carried out, focusing on the functional groups that benefited from an increase in antiproliferative activity in the compounds derived from pyridine and the cancer cell lines that were susceptible to these compounds, as well as those functional groups that were related to a decrease in the antiproliferative activity of the derived compounds and the cancer cell lines in which this situation was seen. Below are more details of the results obtained in this review.

### 2.1. HeLa Cell Line

This is the oldest and most widely used cell line in research, corresponding to cervical and uterine cancer cells [67]. Ninety-nine percent of cases of this type of cancer are related to human papillomavirus (HPV) infections, which are transmitted through sexual contact. Cervical and uterine cancers are the fourth most common cancers in women worldwide, so the World Health Organization (WHO) focuses on preventing them through the use of HPV vaccines and by treating precancerous lesions [68].

Through the structure–activity relationship analysis (SAR) of the reported molecules using this cell line, it was found that the antiproliferative activity is related to the number and position of the O-CH_3_ (OMe) groups. The insertion of NH_2_ and OH groups, halogens (Br, Cl, F), and five- and six-carbon rings also affects the antiproliferative activity of the derived compounds, which is reflected in the IC_50_ values.

When adding OMe groups to a compound, research shows that increasing the number of substituents leads to a decrease in the IC_50_ value, indicating increased activity. For instance, in a study by Zheng et al. (2014) [58], derivative **1** with two OMe groups showed an IC_50_ value of >50 μM, while derivative **2** with three OMe groups had an IC_50_ value of 12 μM. The trend continued when creating more extensive derivatives by inserting six-membered rings. Derivative **3**, with four OMe groups in its structure, recorded an IC_50_ value of <25 μM. Adding two more OMe groups to create derivative **4** further decreased the IC_50_ value to 1.0 μM (as shown in Table 1).

It has been observed that substituting OMe groups with OH groups can significantly decrease the IC_50_ values, which was evident when comparing derivative **5** (with an IC_50_ value of 8 mM) with derivative **6** (with an IC_50_ value of 0.86 mM), where one OMe group was replaced by an OH group [38]. This information is shown in Table 1.

Moreover, Verga et al. (2015) [61] have reported two large compounds that have antiproliferative activity against HeLa, formed by rings of five and six carbons. Derivative **7**, with three rings of six carbons, has a very high IC_50_ value (257 nM), while derivative **8**, with four rings of six carbons, has a much lower IC_50_ value (134 nM). This suggests that the presence of six-carbon rings may lead to a decrease in the IC_50_ values. Table 2 provides more information on these compounds.

El-Sayed et al. (2021) [66] reported two derivatives, **9** and **10** (Table 3), with different substituents at the same site. Derivative **9** had a five-carbon ring with a halogen atom (sulfur) and a high IC_50_ value of 1391 nM, while derivative **10** had a six-carbon ring and chlorine as a halogen, and its IC_50_ value was only 127 nM. These effects were observed in other cell lines, including U937 (myeloid leukemia cells), SKMEL-28 (melanoma), N CIH 460 (lung cancer), and RKOP 27 (colon adenocarcinoma).

Derivative **9** displayed lower IC_50_ values of 422, 255, 25, and 16 nM, respectively. However, it should be noted that this molecule’s impact may vary according to the cell line to which it is exposed, as evidenced by the IC_50_ values ranging from 16 to 422 nM. Therefore, we can infer that each molecule acts differently depending on the specific cell line that it encounters (Table 3) [66].

It is also interesting to note that introducing NH_2_ groups can have an inverse effect on derivatives with rings. For instance, in derivative **11**, which has a five-carbon ring and an ethylenediamine group, the IC_50_ value is 211 nM. However, in compound **12**, which has a six-carbon ring and chlorine, the IC_50_ value increases to 1159 nM (Table 3). An inverse effect can also be observed if the NH group is closer to the rings. This means that if it presents a low IC_50_ value, it increases it and vice versa. This phenomenon is evident in compounds **13** (IC_50_: 1265 nM) and 14 (IC_50_: 255 nM) [66].

### 2.2. A549 Cell Line

This cell line corresponds to human basal alveolar epithelial cells (lung cancer). This type of cancer is the leading cause of cancer-related deaths worldwide, with the highest mortality in both men and women, representing a severe public health problem since it is usually a silent disease in its early stages, which delays its detection [69,70,71].

During the study of derivatives with antiproliferative properties against a specific cell line, it was observed that the compounds’ size and polarity significantly impacted their IC_50_ values. Zheng et al. (2014) [58] found that the CH_3_ groups in the reported derivatives decreased the polarity and IC_50_ values. Conversely, replacing these groups with H atoms increased the polarity and IC_50_ values. A CN group substitution resulted in lower polarity and decreased IC_50_ values, while COOEt replacement increased the polarity and IC_50_ values. For instance, derivative **15** with CH_3_ and COOEt substitutions presented lower IC_50_ values (0.18 mM), whereas derivative **16** with H and a CN group showed higher IC_50_ values (21.05 mM) (Table 1) [58].

It has been observed that the introduction of OMe groups in specific positions reduces the polarity. As a result, derivative **17**, which has an OMe group, exhibits a low IC_50_ value (0.044 mM) compared to derivative **18**, which has an H atom instead of an OMe group. This makes derivative 18 more polar and results in a higher IC_50_ value (4.22 mM) (Table 1).

Regarding the size, larger derivatives, such as derivatives **7** and **8**, with polar surface areas of 83.998 and 88.353, respectively, have higher IC_50_ values of 2708 and 3950 mM, respectively (Table 2). These derivatives have shown similar behavior against other cancer cells, such as epidermoid cancer (A431), glioblastoma (U87), kidney cancer (Hek293), melanoma (A375, M21, and M21L), and lung cancer (H358), exhibiting relatively high IC50 values ranging from 246 to 7700 nM [61].

### 2.3. MCF7 Cell Line

Breast cancer is considered the most prevalent type of cancer worldwide, according to the WHO, affecting women of any age, from puberty onwards, with adult women being the most affected. In 2020, 2.3 million new cases were diagnosed, and 685,000 patients died from this disease. Between 0.5 and 1% of men can be affected by this type of cancer, with the main risk factors being age, obesity, alcohol and tobacco consumption, and family history, among others. The WHO maintains a global initiative against this disease, seeking to reduce breast cancer mortality by 2.5% per year [72].

MCF7 cells are breast adenocarcinoma cells with a low risk of malignancy. They are widely used in cancer biology and research. Zhang et al. conducted antiproliferative activity studies using this cell line. The results showed that the insertion of OH groups reduced the IC_50_ values, as observed in derivative **19** (IC_50_: 4.75 mM), and there was a more significant reduction when inserting two OH groups, as observed in derivative **20** (IC_50_: 0.91 mM). When combining the insertion of OH groups with OMe groups, as in derivative **21**, there was an increase in the IC_50_ value (IC_50_: 3.78 mM). However, the IC_50_ value increased significantly when combining OH groups with halogens such as Cl and Br, as seen in derivatives **22** (IC_50_: 5.83 mM) and **23** (IC_50_: 7.77 mM) (Table 4) [57].

When comparing the IC_50_ values of derivatives with the insertion of OH groups (derivative **19**, IC_50_: 4.75 mM), it was found that the single insertion of an OMe group, a halogen such as fluorine, a NO_2_ group, or a ring resulted in an increase in the IC_50_ values. This can be observed in derivatives **24**, **25**, **26**, and **27**, which have IC_50_ values of 17.63, 24.89, 35.5, and 23.54 mM, respectively (Table 4) [57].

Moreover, large-sized derivatives formed mainly by carbon rings have been reported to have high IC_50_ values [53]. Hassan et al. (2021) [67] also reported higher IC_50_ values for the relatively large derivative **28** (IC_50_: 28.2 mM). However, upon increasing the size of this derivative by inserting more rings and certain groups such as H, CN, OMe, and halogens (fluorine, chlorine, bromine), the IC_50_ values decreased. This can be seen in derivatives **29** (IC_50_: 10.6 mM) and **30** (IC_50_: 0.93 mM) (Table 4).

### 2.4. HepG2 Cell Line

Liver cancer is a more common disease than generally considered. Around the world, over 800,000 people are diagnosed with some form of this cancer every year, and more than 700,000 people die from it. As a result, it is one of the leading causes of cancer-related deaths. Although the most affected populations are found in Asia and Africa, in Mexico alone, there were 4300 new diagnoses, with a mortality rate of 4000 [73,74], in the year 2020.

The HepG2 cell line is used to study liver cancer’s metabolism and pharmacological toxicity. Sangani et al., in 2014, reported that certain derivatives have antiproliferative activity against this cell line. Derivative **31**, which has a single CH_3_ group attached to an aromatic ring, has an IC_50_ value of 1.30 mM. Derivatives **32** and **33**, which replace the CH_3_ group with Cl and H, respectively, have higher IC_50_ values of 5.84 mM and 7.15 mM (Table 5). The trend continues with derivatives **34**, **35**, and **36**, where inserting two CH_3_ groups in place of the two H atoms attached to an aromatic ring results in IC_50_ values of 4.04 mM, 6.21 mM, and 24.4 mM, respectively (Table 5). These data show that the antiproliferative activity is favored by inserting a CH_3_ group, followed by H and then Cl. This may be due to the larger area, volume, and polar surface of the derivatives that present the CH3 group, as the molecular descriptors indicate. The derivative that possesses H is more significant than the one presenting Cl in the absence of the CH_3_ group [56].

Sangani et al. (2014) also reported that when the COOEt group was changed to a CN group in derivatives **34**, **35**, and **36**, it resulted in derivatives **37**, **38**, and **39**. In **37** (IC_50_: 1.02 mM), the IC_50_ value decreased compared to its counterpart, **35**, while, in **38** (IC_50_: 4.83 mM) and **39** (IC_50_: 15.17 mM), the IC_50_ value increased compared to their counterparts, **34** and **36**, respectively (as shown in Table 5). The changes in the IC_50_ values may have been due to the insertion of the CN group, which led to increased HOMO and LUMO energy values and decreased CPKA and CPKV values, as indicated in Table 6 [56].

Zhang et al. (2014) found that inserting halogens such as fluorine, chlorine, bromine, NO_2_, OMe, or CH_3_ in a ring’s ortho, meta, or para positions did not significantly alter the IC_50_ values. The derivatives **40**, **41**, **42**, and **43** had IC_50_ values of 12.3 mM, 24.7 mM, 15.84 mM, and 10.02 mM, respectively. However, adding an OH group in the para or ortho position caused a significant decrease in the IC_50_ values, as seen in derivatives **44** (IC_50_: 5.51 mM) and **19** (IC_50_: 3.51 mM). They also observed that inserting a halogen (Cl) in the same ring produced derivative **22** (IC_50_: 3.97 mM), but the IC_50_ value did not change substantially. Finally, they inserted an extra OH group and obtained their best result with derivative **20**, with an IC_50_ value of 0.76 mM (Table 4) [57].

### 2.5. Cell Lines Hep2 and PC3

The Hep2 and PC3 cell lines correspond to epidermoid and prostate carcinoma, respectively. Skin cancer is the most common type of cancer worldwide, but it has a low mortality rate [68]. In contrast, prostate cancer represents a serious public health issue, being the most commonly occurring type of cancer in men and the fifth leading cause of death worldwide. In the United States of America alone, it is estimated that more than 299,010 new cases of prostate cancer will be diagnosed in 2024, with almost 35,000 deaths being reported due to this disease [75,76,77].

Alqahtani and Bayazeed (2020) [65] have found that adding a COOEt group to the five-carbon ring of derivative **45** reduces the IC_50_ values (Hep2 IC_50_: 17.71 mM; PC3 IC_50_: 18.36 mM) (Table 5). This is due to the increased molecular area and volume and, more significantly, the large increase in the polar surface area (Table 6). Conversely, adding aromatic rings or aliphatic chains increases the IC_50_ values. This can be observed in molecules **46** (Hep2 IC_50_: 43.36 mM; PC3 IC_50_: 37.17 mM) and **47** (Hep2 IC_50_: 37.44 mM; PC3 IC_50_: 42.31 mM) (Table 6). Similar results were observed for derivatives tested on the MCF-7 and HepG2 cell lines [65].

### 2.6. Cell Lines SW1116 and BGC823

Zhang et al. (2014) [57] studied the cell lines associated with colon and gastric cancer. Colon or colorectal cancer is the third most prevalent cancer globally and the second most common cause of cancer-related deaths. It primarily affects older adults and is linked to unhealthy lifestyles, such as the high consumption of processed meats, low intake of fruits and vegetables, sedentary lifestyles, obesity, alcohol consumption, and smoking [71]. Stomach cancer predominantly affects older people, especially women. Only 1.5% of all new cancer cases worldwide are estimated to be due to stomach cancer [78].

The MCF7 and HepG2 cell lines show that inserting two OH groups produces the best results, resulting in derivative **20** (SW1116 IC_50_: 1.54 mM; BGC823 IC_50_: 1.26 mM). It is also observed that when halogens are inserted, the IC_50_ values increase in proportion to the size of the halogen, with the smallest being fluorine (42 pm), followed by chlorine (175 pm) and, finally, bromine (185 pm). This can be seen by comparing derivatives **40** (SW1116 IC_50_: 15.71 mM; BGC823 IC_50_: 17.84 mM), **48** (SW1116 IC_50_: 17.02 mM; BGC823 IC_50_: 20.52 mM), and **49** (SW1116 IC_50_: 21.32 mM; BGC823 IC_50_: 24.51 mM) (Table 4). Additionally, when comparing the IC_50_ values obtained by inserting the smallest halogen, fluorine, it is observed that the para position corresponding to derivative **40** generates a lower IC_50_ value than the target position, derivative **25** [57].

### 2.7. DLA Cell Line

Ascites are usually a consequence of some types of cancer that have reached more advanced stages by spreading to the abdominal area, such as the ovary, liver, colon, stomach, and pancreas [79].

Al-Gorbani et al. (2016) [60] reported some derivatives with antiproliferative activity against Dalton lymphoma ascites (DLA) cells. The study found that when the derivatives included OH groups and halogens, there was a decrease in the IC_50_ values. The molecules that showed the most significant reductions in the IC_50_ values were **50** (IC_50_: 9 mM), **51** (IC_50_: 8 mM), and **52** (IC_50_: 40 mM) (Table 7). The size of the halogen also played a role in the IC_50_ values, with the smallest halogens, F and Cl, showing the lowest IC_50_ values, while I, the largest halogen, showed the highest IC_50_ value [60].

It is important to note that in cases where the suggested modifications involved adding two halogens or a halogen and an NH group, the IC_50_ values increased, leading to decreased antiproliferative activity. This is due to a reduction in the polar surface of such derivatives, as seen in derivatives **53** (IC_50_: 45 mM) and **54** (IC_50_: 65 mM) [60].

### 2.8. MDA-MB-231 Cell Line

One of the most widely used breast cancer cell lines in cancer research is MDA-MB-231, which is highly invasive. Zhang et al. (2014) reported derivatives with antiproliferative activity against this cell line. The behavior of these derivatives was similar to that of the A549 and HeLa cell lines. The IC_50_ values decreased in the derivatives where OMe groups were inserted, such as in derivatives **55** (IC_50_: 9.0 mM) and **56** (IC_50_: 0.075 mM). Combining OMe and OH groups in the derivatives also decreased the IC_50_ values. For example, derivatives **57** (CI_50_: 0.069 mM) and **58** (CI_50_: 0.0046 mM) (Table 4) showed a decrease in their IC_50_ values. However, including OH groups in the derivatives increased the PSA, decreasing the IC_50_ values [57].

Chen et al. (2021) [63] conducted research using two mammary carcinoma lines, MDA-MB-453 and SK-BR-3. Their report suggests that inserting CH_3_ and NO_2_ groups in the para position improves the antiproliferative activity by decreasing the IC_50_ values. Derivatives **59** and **60** are examples, with IC_50_ values of 4.9 nM and 9.0 nM, respectively. However, in the case of the CH_3_ group, substitutions in the ortho and meta positions increase the IC_50_ values. Derivatives **61** and **62** are examples, with IC_50_ values of 91.9 nM and 82.4 nM, respectively. The insertion of OH groups also increases the IC_50_ values, as seen in derivatives **63** and **64**, with IC_50_ values of 27.7 nM and 41.4 nM, respectively. Similarly, the insertion of NH_2_ and OMe groups in the para positions increases the IC_50_ values, as observed in derivatives **65** and **66**, with IC_50_ values of 27.1 nM and 50.7 nM, respectively.

However, inserting two combined groups in the meta–para positions, such as OH and CH_3_ or NH_2_ and CH_3_, generates better results in terms of the antiproliferative activity. Derivatives **67** and **68** are examples, with IC_50_ values of 1.7 nM and 2.8 nM, respectively [63].

The derivatives reported by Chen et al. (2021) for breast cancer cells exhibit similar behavior to that observed for other melanoma cell lines—specifically the A375, M14, and RPMI 7951 cell lines. Among these derivatives, derivative **67** showed the best results, with IC_50_ values of 1.5 nM, 1.7 nM, and 1.7 nM for A375, M14, and RPMI 7951, respectively (Table 8). Derivative **67** is identical in structure, with the only difference being the insertion of an OH and CH_3_ group, increasing its polarity [63].

### 2.9. Analysis of Electrostatic Potential Maps in Most Promising Derivatives

Electrostatic potential maps (EPMs) are powerful tools with which to understand the relationship between a molecule’s electron density and its biological activity. They can help to predict and explain non-covalent interactions between ligands and target molecules. EPMs use different colors to represent regions of varying electron density within a molecule. Low-electron-density regions are shown in blue, high-density regions in red, and green areas represent regions with a zero EPM value that are prone to hydrophobic interactions [80,81].

**Figure 2:**
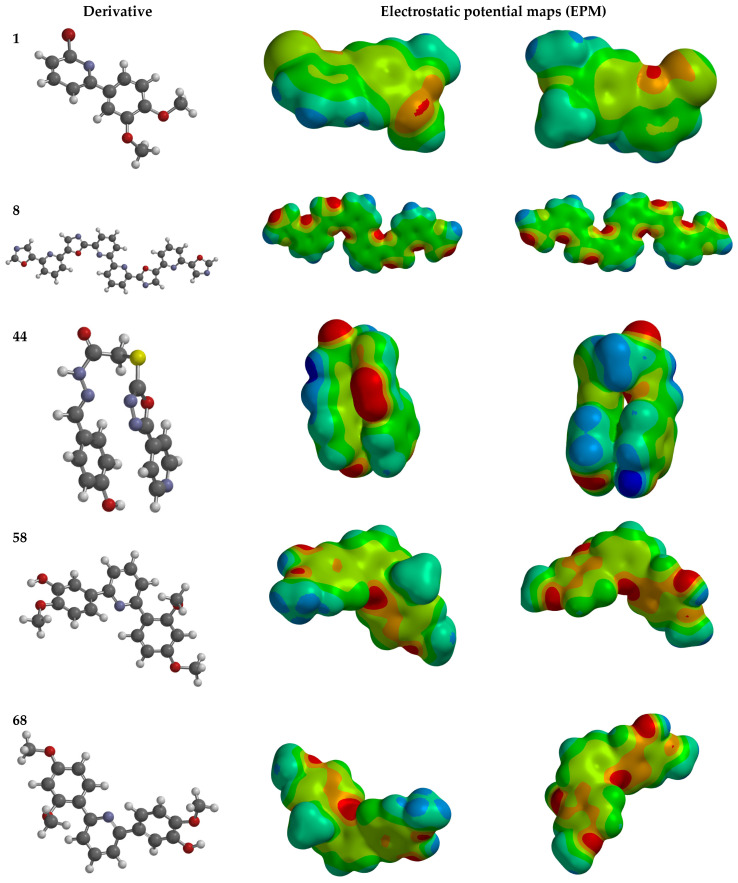
Electrostatic potential maps of pyridine derivatives containing OMe, OH, bromine, CH_3_, NO_2_, and aromatic ring groups. Zero, negative, and positive values of EPM are depicted as green, red, and blue colored regions, respectively.

An analysis of the electrostatic potential maps (Figure 2) was carried out to establish a correlation between the chemical structures of certain compounds and their biological activity. This analysis focused on compounds that showed lower values of IC_50_ in their antiproliferative activity. The optimized structures of these compounds were used to study regions with high and low electron densities in their ground states. The compounds subjected to the EPM analysis were **1**, **8**, **44**, **58**, and **68**.

After analyzing the EPMs and structures of the selected compounds, it was observed that groups containing electronegative elements such as O and N had a higher electron density (red regions). For instance, in compound **68**, red areas were observed in the EPMs, where methoxyl groups (O-CH_3_) were bound to the aromatic and carbonyl rings (C=O). Blue zones (lower electron density) were found in the amino group (-NH_2_) bound to the aromatic ring. In compound **8**, the red zone was shown in the nitrogen atoms of the oxazole groups of the molecule, while the oxygen atoms of the same groups showed an orange zone of lower electron density. Furthermore, the blue regions were found in the -CH groups of the pyridine rings. Compound **58** showed few zones of high and low electron density, but it is noteworthy that the compound contains a -OH group close to an -OCH_3_ group in one of the aromatic rings. This group has two main zones: the red zone positioning itself in the oxygen atom and a blue zone in the H atom. Lastly, the EPM of compound **44** showed that the red zone was placed in the carbonyl, hydroxyl, and nitrogen of the 1,3,4-oxadiazole ring.

These compounds have large green areas, representing neutral regions of zero electron density. These regions are identified in the EPM as being prone to the formation of hydrophobic interactions.

## 3. Discussion

This work is a significant compilation of antiproliferative activity studies using pyridine-derived compounds against various human cancer cell lines. The aim was to conduct a comprehensive analysis of the structure–activity relationships of these compounds, as reported in various articles published from 2012 to 2022. The objective was to identify the most effective chemical modifications that enhance the biological activity of these compounds.

Pyridine, a nitrogen-containing heterocycle compound, is the second most common heterocycle-type compound approved by the FDA. Its diverse biological activity, including antituberculosis, antitumor, antimalarial, and antimicrobial activity, has been extensively studied. Given its chemical structure and these properties, it serves as an innovative template for the design and synthesis of new compounds with enhanced biological activity.

The analysis of the antiproliferative structure–activity relationships of various pyridine derivatives has yielded significant findings. These findings can potentially guide the design of new antitumor drugs based on pyridine as the main scaffold. We will present the key results obtained in different human cancer cell lines and discuss how specific chemical modifications either enhance or hinder the efficiency of these compounds.

**Figure 3:**
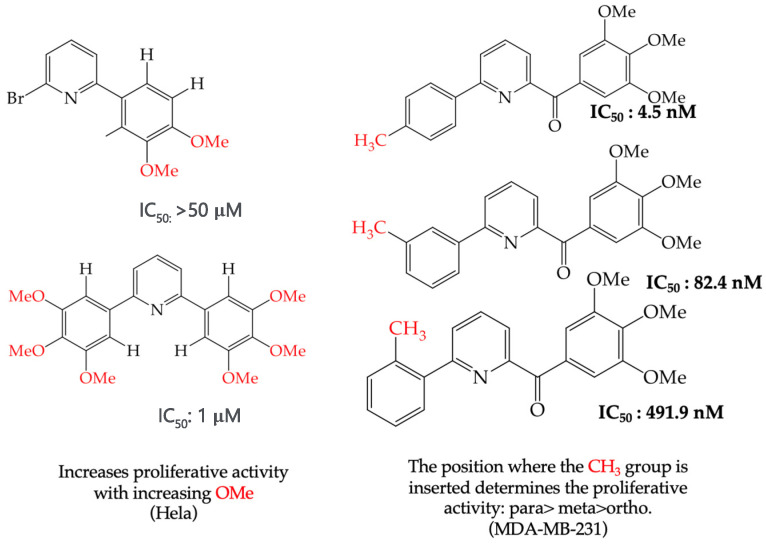
Examples of compounds derived from pyridine where antiproliferative activity and various functional groups are related to OMe and CH_3_.

**Figure 4:**
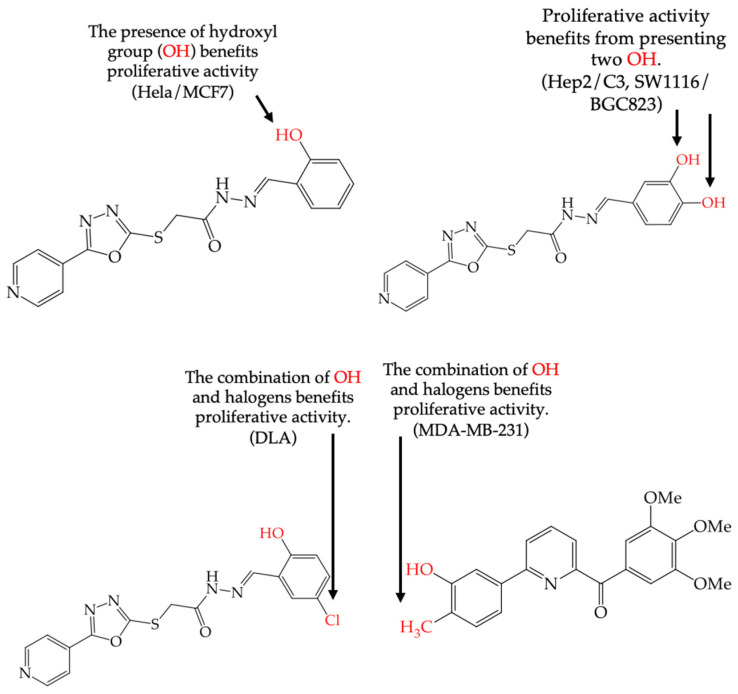
Examples of compounds derived from pyridine where antiproliferative activity and various functional groups are related to OH.

The introduction of O-CH_3_ groups in the structure of the pyridine-derived compounds significantly improved their antiproliferative activity, mainly against the HeLa, A549, and MDA-MB-231 cell lines (Figure 3), observing a decrease in the IC_50_ values; this indicates an improvement in antiproliferative activity, impacting the effectiveness in inhibiting cell growth against these cell lines. Another functional group that favored an improvement in the antiproliferative activity of the pyridine-derived compounds was the -OH group, obtaining lower IC_50_ values, mainly in the HeLa and MCF-7 cell lines (Figure 4). In addition, it was observed that when associated with the structure of the compound with two -OH groups, the IC_50_ values were better, mainly regarding the antiproliferative activity against the cell lines Hep2, PC3, SW1116, and BGC823.

Currently, we can find several studies on the search for and design of drugs to analyze the structure–activity relationships and determine which chemical modifications favor the biological activity of certain compounds. Miladiyah et al. (2018) [82] carried out a QSAR study of several compounds derived from xanthones, where they demonstrated that the functional groups that showed the best antiproliferative activity were those that presented electron donor atoms, such as OHOH (IC50 = 0.019 μM), OCH_3_ (IC_50_ = 0.058 μM), OCOCH_3_ (IC_50_ = 0.035 μM), and NHCOCH_3_ (IC_50_ = 0.021 μM).

Using the chemical structures of compounds derived from natural products is a fascinating strategy for the design of compounds with better antiproliferative activity against various cancer cell lines. Saito et al. (2015) [83] developed various compounds derived from chalcones, where different substituents were added, and the compounds obtained were evaluated against the HL60 cell line (human promyelocytic leukemia cells). The results demonstrated that the OH and OMe substituents included in the chalcones as a central scaffold showed a better IC_50_ value, obtaining values between 12.3 and 19.9 μM. The authors concluded that not only were these substituents related to the activity shown by the chalcone-derived compounds but the spatial region in the chemical structure influenced the activity.

Another group that favored the activity of the compounds studied was the -CH_3_ group. By introducing a small non-polar group into the structure, low IC50 values were observed in the results, mainly in the A549 and HepG2 cell lines

**Figure 5:**
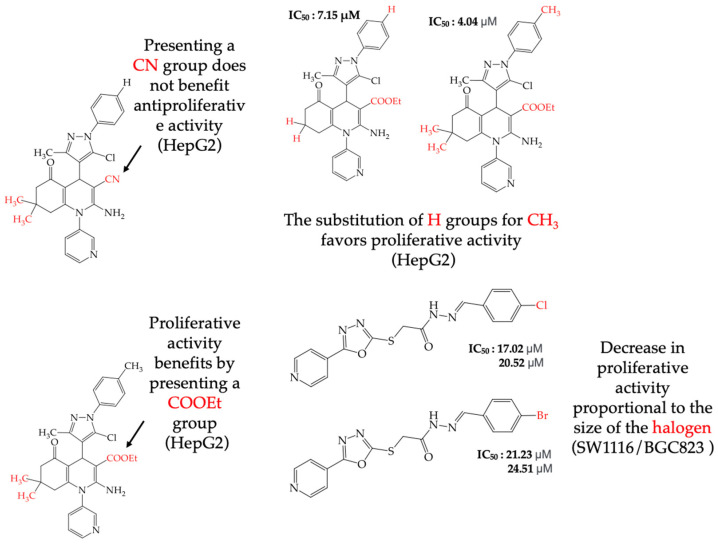
Examples of compounds derived from pyridine where antiproliferative activity and various functional groups are related to CN, COOEt, CH_3_, H, and halogens.

Different studies reporting on compounds derived from pyridine agreed that introducing some functional groups affected their antiproliferative activity, mainly halogens and -CN. In the case of introducing halogens (Cl, F, Br), an increase in the IC_50_ values was observed, but this depended on two main characteristics of these elements: their size (the more significant the atom, the higher the IC_50_ value) and their electrophilic capacity. The behavior of the cyano group (-CN) in the different derivatives was variable; an increase in the IC_50_ value was observed in the activity against the A549 cell line, and, in some compounds, an increase in the IC_50_ value was observed, while, in other cases, a decrease in activity against the HepG2 cell line was noted. The carboxylic ethyl ester group (COOEt) had similar behavior to the cyano group, where a variation in the IC_50_ values was observed, showing favorable values for the antiproliferative activity in the Hep2 and PC3 cell lines (Figure 5).

Cedrón et al. (2015) [84] made various modifications to alkaloid compounds, obtaining various derived compounds and evaluating their antiproliferative activity against two cancer cell lines: A2780 (ovary), SW1573 (lung), T47-D (breast), and WiDr (colon). From the results obtained in this study, the authors conclude that the OH groups in the structures of these alkaloids are of great importance for their antiproliferative activity. They observed that when replacing this group with Cl and OMe, the activity decreased, showing IC_50_ values between 4.3 and 8.3 μM before the modification and IC_50_ values between 75 and 100 μM after the modification. They also observed that the acylation of the OH in these derived compounds decreased their antiproliferative activity, concluding that the presence of H donor groups in the compounds benefits their biological activity.

This analysis of the antiproliferative activity of pyridine-derived compounds shows that these compounds have promising potential when directed against the cancer cell lines evaluated. Some studies have investigated the characteristics of both cells and compounds that may influence the behavior of these derived compounds, mentioning that the inhibition of cell growth, which affects cells differently depending on the type, depends on the nature of the compound, as well as the structure and organization of the membrane [58].

## 4. Materials and Methods

**Figure 6:**
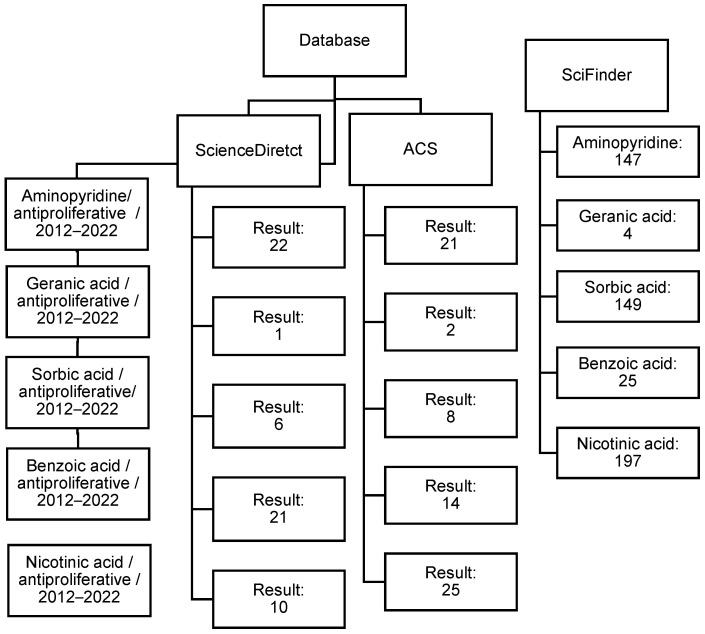
Flowchart depicting the process of selecting relevant articles for this review.

A search was conducted for articles published between 2012 and 2022. The search considered articles that reported information on pyridine derivatives and carboxylic-acid-type compounds such as benzoic acid, sorbic acid, nicotinic acid, and geranic acid. These articles included the antiproliferative activity, reported in terms of IC_50_ values, as part of the biological evaluation. The review was carried out mainly in three databases, ScienceDirect, SciFinder, and the American Chemical Society (ACS), and encompassed 652 articles; see Figure 6.

After the initial search, it was decided to narrow the scope of the review to articles that reported pyridine derivatives with antiproliferative activity. Only ten papers, which collectively reported more than 200 derivatives, met the defined criteria for this review.

Subsequently, each derivative was constructed using the ChemDraw Professional 20.0.0.41 (Revvity Signals Software, Inc., Waltham, MA, USA, 2023). Spartan ‘18 (Wavefunction Inc., Irvine, CA, USA, 2018) was used to perform the conformational analysis, with a molecular mechanics level of theory with the Merck Molecular Force Field (MMFF). The minimum energy conformation of each derivative was selected for geometry optimization using the semi-empirical method parametric method number 6 (PM6). The generated information was used to calculate the various molecular descriptors. These descriptors included the total molecular volume area (CPK area and CPK volume) from the family of constitutional descriptors, the polar surface area (PSA) from the molecular properties family, and some descriptors from the quantum mechanics family, such as the energy of the highest-energy occupied orbital (E_Homo_) and the energy of the lowest-energy unoccupied orbital (E_Lumo_). These analyses were performed to understand the behavior of the derivatives better.

## 5. Conclusions

The chemical and biological characteristics of the pyridine structure have been a subject of significant interest in research for the development of new drugs. The structure acts as a structural scaffold for the creation of novel molecules.

In the analysis performed herein, it was discovered that compounds containing groups with nitrogen and oxygen in their structure, such as -OCH_3_, -OH, -C=O, and NH_2_, either alone or included in aromatic rings, oxazoles, and pyrimidines, exhibited superior antiproliferative activity against various human cancer cell lines. The study conducted through the EPMs revealed that interactions with the studied cells can occur in these regions. It was also observed that these pyridine-derived compounds exhibited green primary areas in the EPMs, which represented hydrophobic zones that can provide more significant interactions with the cell membrane. This interaction helps the compound to enter the cell’s interior, interact more easily with the cellular components, and carry out its activity of inhibiting cell growth.

## Figures and Tables

**Table 1:** Derivatives reported by Zheng et al. (2014) [58].

Derivative	Cell Line	IC_50_ Value	Molecular Descriptors				
			E_Homo_	E_Lumo_	CPK_área_	CPK_volumen_	PSA
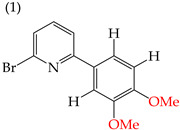	HeLa	>50 μM	−8.99	−0.65	272.34	249.43	21.099
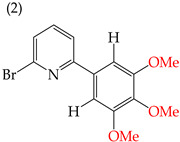	HeLa	12 μM	−9.15	−0.77	303.82	277.07	23.394
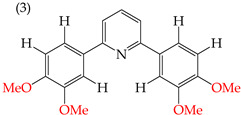	HeLa	<25 μM	−8.91	−0.073	392.96	396.66	34.545
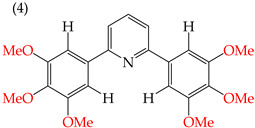	HeLa	1 μM	−8.95	−0.88	455.43	424.88	49.309
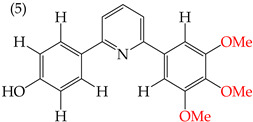	HeLa	8 μM	−8.32	−0.52	340.33	323.4	37.418
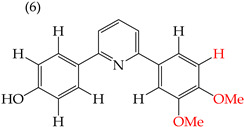	HeLa	0.86 μM	−8.32	−0.52	340.33	323.4	37.418
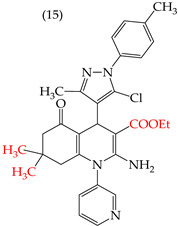	A549	0.18 μM	−8.59	−0.71	554.04	543.31	69.085
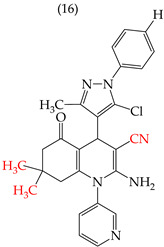	A549	21.05 μM	−8.81	−1.26	444.81	444.33	67.838
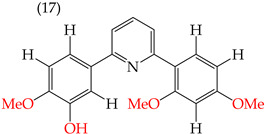	A549	0.044 μM	−8.4	−0.29	369.20	350.40	42.760
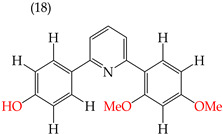	A549	4.22 μM	−8.51	−0.27	339.73	322.94	37.286

**Table 2:** Derivatives reported by Verga et al. (2015) [61].

Derivative	Cell Line	IC_50_ Value	Molecular Descriptors				
			E_Homo_	E_Lumo_	CPK _área_	CPK _volumen_	PSA
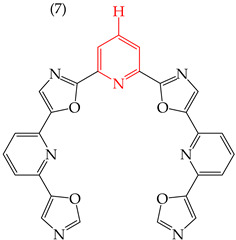	HeLa	257 nM	−8.90	−1.34	500.95	477.20	83.998
	A549	2708 μM					
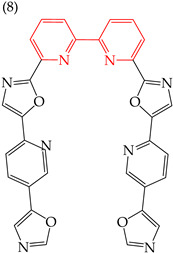	HeLa	134 nM	−9.02	−1.30	578.77	554.38	88.353
	A549	3950 μM					

**Table 3:** Derivatives reported by El-Sayed et al. (2021) [66].

Derivate	Cell Line	IC_50_	Molecular Descriptors				
			E_Homo_	E_Lumo_	CPK _área_	CPK _volumen_	PSA
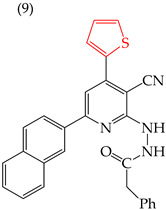	HeLa	1391 nM	−8.68	−1.17	481.18	467.14	57.748
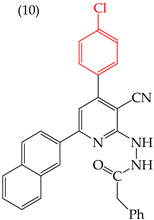	HeLa	127 nM	−8.76	−1.17	507.85	493.35	58.011
	U937	422 nM					
	SKMEL-28	255 nM					
	N CIH 460	25 nM					
	RKOP 27	16 nM					
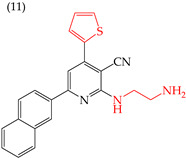	HeLa	211 nM	−8.88	−1.02	391.45	378.41	58.958
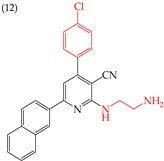	HeLa	1159 nM	−8.95	−1.01	417.96	404.59	58.993
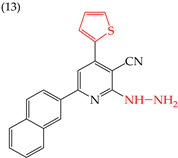	HeLa	1265 nM	−9.02	−1.21	352.62	341.04	61.868
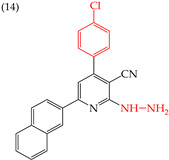	HeLa	255 nM	−9.05	−1.22	380.14	367.45	62.783

**Table 4:** Zhang et al.’s (2014) [57] reported derivatives and the values of the molecular descriptors obtained for each molecule.

Derivative	Cell Line	IC_50_ Value	Molecular Descriptors				
			E_Homo_	E_Lumo_	CPK _área_	CPK _volumen_	PSA
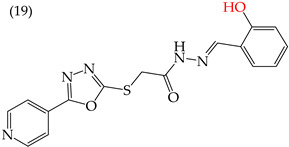	MCF7	4.75 μM	−9.18	−1.60	340.00	329.32	86.166
	HepG2	3.51 μM					
	SW1116	7.38 μM					
	BGC823	6.16 μM					
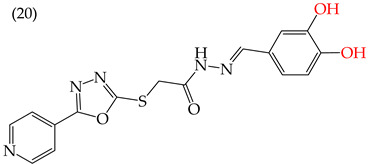	MCF7	0.91 μM	−8.84	−1.54	358.47	338.01	103.73
	HepG2	0.76 μM					
	SW1116	1.54 μM					
	BGC823	4.32 μM					
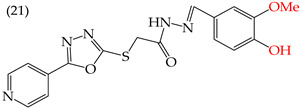	MCF7	3.78 μM	−8.73	−1.52	378.62	357.9	93.305
	SW1116	3.59 μM					
	BGC823	1.26 μM					
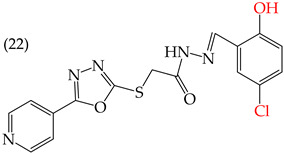	MCF7	5.83 μM	−9.24	−1.69	351.33	342.49	86.284
	HepG2	3.97 μM					
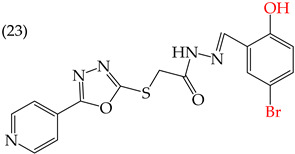	MCF7	7.77 μM	−8.75	−1.15	372.06	345.85	84.591
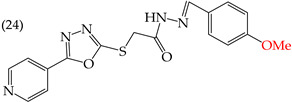	MCF7	17.63 μM	−9.85	−1.43	372.20	350.78	76.033
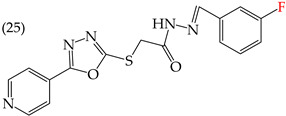	MCF7	24.89 μM	−9.55	−1.53	348.70	328.07	68.727
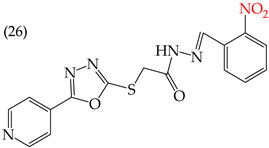	MCF7	35.5 μM	−9.75	−1.72	364.39	344.42	104.106
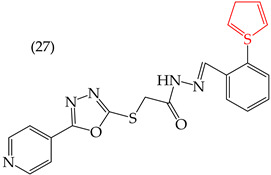	MCF7	23.54 μM	−7.74	−1.59	410.63	400.06	69.399
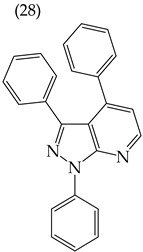	MCF7	28.2 μM	−8.61	0.62	371.98	371.93	16.836
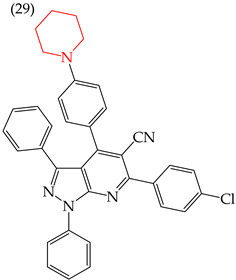	MCF7	10.6 μM	−8.11	−0.99	570.61	578.85	32.696
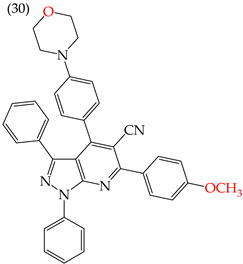	MCF7	0.93 μM	−8.21	−0.89	577.72	584.21	47.340
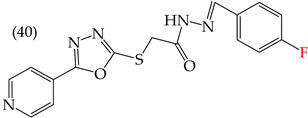	HepG2	12.3 μM	−9.43	−1.51	351.69	328.26	68.847
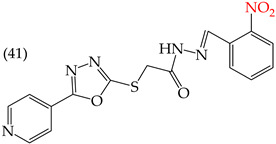	HepG2	24.7 μM	−9.75	−1.72	364.39	344.42	104.106
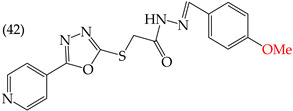	HepG2	15.84 μM	−8.85	−1.43	372.20	330.78	76.033
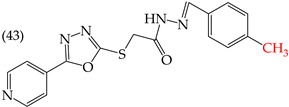	HepG2	10.02 μM	−9.11	−1.42	362.07	341.54	69.231
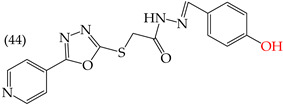	HepG2	5.51 μM	−9.07	−1.58	339.66	329.06	89.252
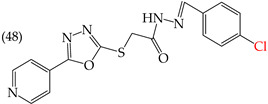	SW1116	17.02 μM	−9.41	−1.48	355.95	336.57	68.587
	BGC823	20.52 μM					
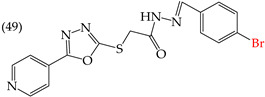	SW1116	21.32 μM	−8.81	−1.04	377.86	342.17	71.378
	BGC823	24.51 μM					
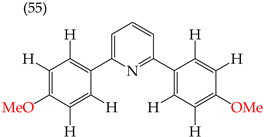	MDA-MB-231	9.0 μM	−8.57	−0.38	333.63	316.35	20.438
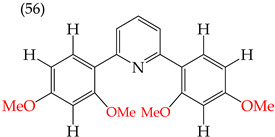	MDA-MB-231	0.075 μM	−8.85	−0.17	391.38	369.01	33.722
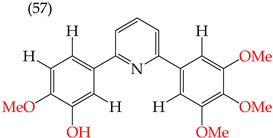	MDA-MB-231	0.069 μM	−8.57	−0.57	398.15	377.90	48.693
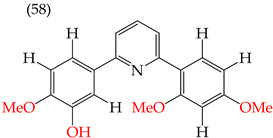	MDA-MB-231	0.0046 μM	−8.40	−0.29	369.20	350.40	42.758

**Table 5:** Derivatives reported by Sangani et al. (2014) [56] and the values of the molecular descriptors obtained for each molecule.

Derivative	IC_50_ Value vs. HepG2	Molecular Descriptors				
		E_Homo_	E_Lumo_	CPK _área_	CPK _volumen_	PSA
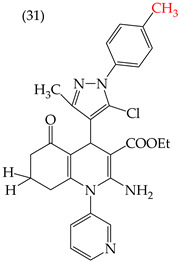	1.30 μM	−8.54	−1.15	511.35	508.92	69.141
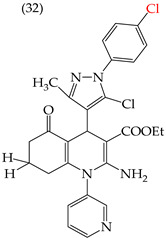	5.84 μM	−8.77	−1.23	506.67	504.23	68.821
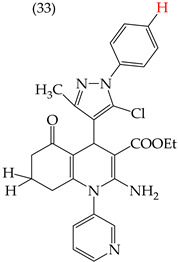	7.15 μM	−8.75	−1.18	493.95	491.02	68.259
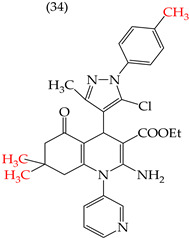	4.04 μM	−8.60	−1.11	544.09	544.11	60.389
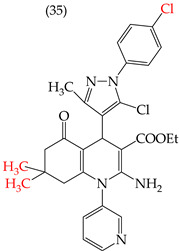	6.21 μM	−8.78	−1.20	539.72	539.43	68.181
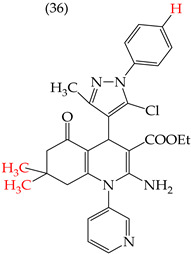	24.4 μM	−8.73	−1.15	520.56	526.33	68.213
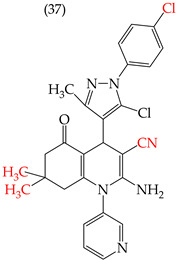	1.02 μM	−8.86	−1.29	495.25	493.4	67.811
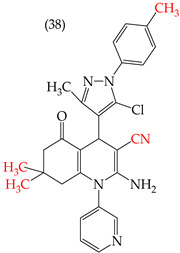	4.83 μM	−8.75	−1.21	499.42	498.03	67.889
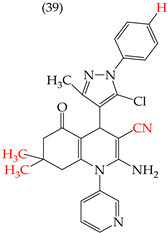	15.17 μM	−8.79	−1.23	479.68	479.89	67.852

**Table 6:** Derivatives reported by Alqahtani and Bayazeed (2020) [65].

Derivative	Cell Line	IC_50_ Value	Molecular Descriptors				
			E_Homo_	E_Lumo_	CPK _área_	CPK _volumen_	PSA
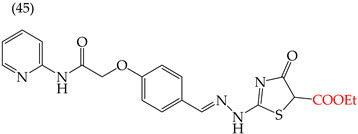	Hep2	17.71 μM	−9.17	−1.28	452.82	416.95	99.324
	PC3	18.36 μM					
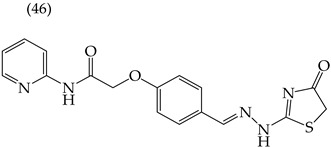	Hep2	43.36 μM	−9.09	−1.14	378.85	349.72	78.567
	PC3	37.17 μM					
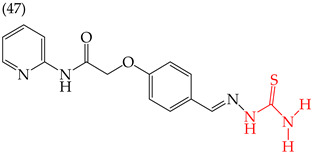	Hep2	37.44 μM	−8.53	−0.81	392.65	358.72	54.54
	PC3	42.31 μM					

**Table 7:** Derivatives reported by Al Gorbani et al. (2016) [60] and the values of the molecular descriptors obtained for each molecule.

Derivative	IC_50_ Value vs. DLA	Molecular Descriptors				
		E_Homo_	E_Lumo_	CPK_área_	CPK_volumen_	PSA
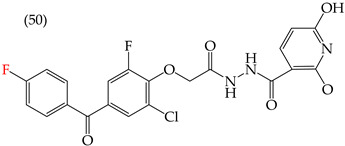	9 μM	−9.73	−1.13	429.81	405.89	98.197
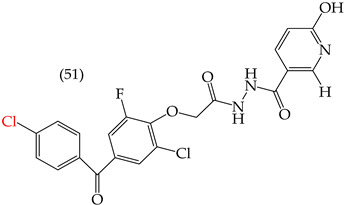	8 μM	−9.73	−1.14	440.28	414.83	98.623
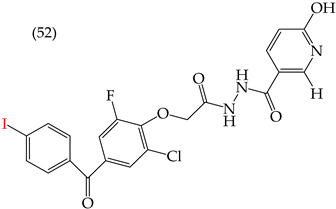	40 μM	−9.22	−0.86	450.64	424.48	101.257
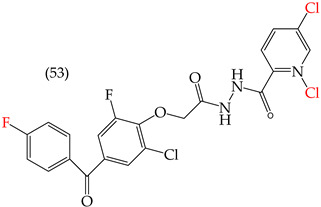	−9.73	−1.14	440.28	414.83	98.623	56.513
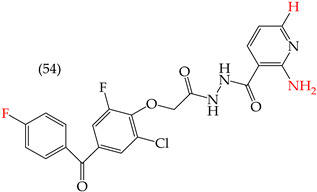	−9.22	−0.86	450.64	424.48	101.257	97.061

**Table 8:** Derivatives reported by Chen et al. (2021) [63] and the values of the molecular descriptors obtained for each molecule.

Derivative	IC_50_ vs. MDA-MB-231	Molecular Descriptors				
		E_Homo_	E_Lumo_	CPK_área_	CPK_volumen_	PSA
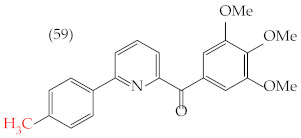	4.9 nM	−8.77	−0.70	402.53	382.49	37.457
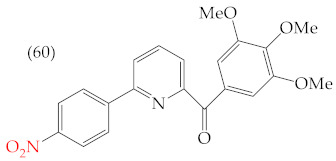	9.0 nM	−8.81	−0.78	391.97	371.67	57.353
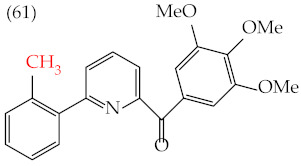	91.9 nM	−8.81	−0.70	399.51	382.07	37.217
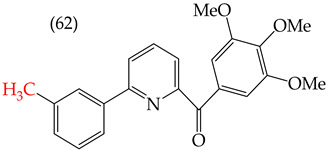	82.4 nM	−8.77	−0.75	402.40	382.46	37.27
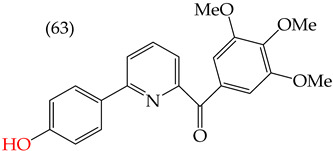	27.7 nM	−8.81	−0.78	391.97	371.67	57.335
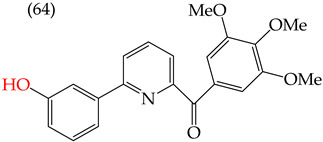	41.4 nM	−8.87	−1.07	387.97	371.32	58.033
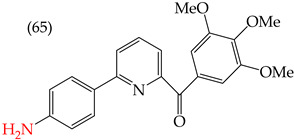	27.1 nM	−8.61	−0.68	396.19	374.84	61.932
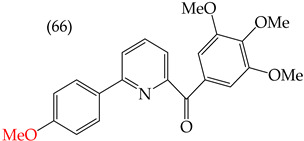	50.7 nM	−8.53	−0.76	14.77	312.87	61.049
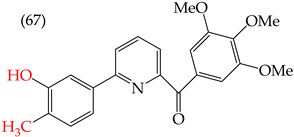	1.7 nM	−8.87	−0.83	410.22	389.39	56.513
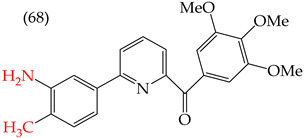	2.8 nM	−8.53	−0.76	414.77	392.87	61.049

## Abbreviations

HeLa	Cervical and uterine cell line
HPV	Human papillomavirus
WHO	World Health Organization
SAR	Structure–activity relationship
OMe	O-CH_3_
U937	Myeloid leukemia cell line
SKMEL-28, A375, M21, and M21L	Melanoma cell line
N CIH	460 and H358 lung cell line
RKOP 27	Colon cell line
A549	Lung cell line
A431	Epidermoid cell line
U87	Glioblastoma cell line
Hek293	Kidney cell line
MCF7	Breast cell line
HepG2	Liver cancer
Hep2	Epidermoid carcinoma cell line
PC3	Prostate carcinoma cell line
SW1116	Colon cancer cell line
BGC823	Gastric cancer cell line
DLA	Dalton lymphoma ascites
MDA-MB-231	Breast cancer cell line
EPM	Electrostatic potential map
O-CH_3_	Methoxyl groups
COOEt	Carboxylic ethyl ester group
(-CN)	Cyano group
MMFF	Merck Molecular Force Field
PM6	Semi-empirical method parametric method number 6
CPKv	Total molecular volume
CPKa	Total molecular area
PSA	Polar surface area
E_Homo_	Energy of the highest-energy occupied orbital
E_Lumo_	Energy of the lowest-energy unoccupied orbital
